# Viral hepatitis associated hepatocellular carcinoma on the African continent, the past, present, and future: a systematic review

**DOI:** 10.1186/s12885-021-08426-y

**Published:** 2021-06-19

**Authors:** Ottovon Bismark Dakurah, Cynthia Raissa Tchuem Tamandjou, Moleen Zunza, Wolfgang Preiser, Tongai Gibson Maponga

**Affiliations:** 1 African Cancer Institute, Department of Global Health, Faculty of Medicine and Health Sciences, Stellenbosch University, Cape Town, South Africa; 2 Division of Health Economics, School of Public Health and Family Medicine, University of Cape Town, Cape Town, South Africa; 3 Division of Epidemiology and Biostatistics, Department of Global Health, Faculty of Medicine and Health Sciences, Stellenbosch University, Cape Town, South Africa; 4 Division of Medical Virology, Faculty of Medicine and Health Sciences, Stellenbosch University, Cape Town, South Africa

**Keywords:** Hepatocellular carcinoma, Hepatitis B virus, Hepatitis C virus, Hepatitis D virus, Africa, Sub-Saharan Africa, Direct-acting antivirals (DAAs)

## Abstract

**Background:**

Hepatocellular carcinoma (HCC) is one of the leading causes of cancer-related deaths in Africa. In Africa, the major causes of HCC include chronic infection with hepatitis B virus (HBV) and/or hepatitis C virus (HCV). Knowledge of the changes in the incidence of viral hepatitis-associated HCC over time and the factors responsible for such changes is key in informing policies for the prevention of viral hepatitis-associated HCC in Africa.

**Aim:**

The study aimed to systematically summarize the changes in the prevalence of viral hepatitis among HCC patients and the overall effect of the prevalence of viral hepatitis on the incidence of HCC over the past four decades in Africa (1980–2019).

**Methods:**

A literature search was conducted in MEDLINE (PubMed), Google Scholar, Science Direct, Scopus, Web of Science, and African wide web for articles published on viral hepatitis-associated HCC in Africa from 1980 to 2019. The abstracts of the articles were screened for eligibility and those meeting the inclusion criteria were retrieved and reviewed.

**Results:**

A total of 272 studies were included in the analysis. Viral hepatitis-related HCC incidence changed by 1.17% (95% confidence interval (CI): 0.63–1.71, *p < 0.001*), 0.82% (95% CI: 0.45–1.18, *p < 0.001*), and 3.34% (95% CI: 2.44–4.25, *p < 0.001*) for every 1% change in the prevalence of HBV, HCV, and hepatitis D virus (HDV) respectively, per decade. The incidence of HBV-related HCC decreased by − 0.50% (95% CI: − 0.74 – − 0.25, *p* < 0.001) over the last 40 years, while HCV-related HCC increased.

**Conclusion:**

Overall, the incidence of viral hepatitis-associated HCC has not declined, mainly due to no decline in the prevalence of HCV, HDV, and the high number of chronic hepatitis B carriers on the African continent. There is an urgent need for the allocation of resources for the implementation of treatment and preventive programs for HBV, HCV, HDV, and HCC in Africa.

This systematic review is registered with PROSPERO®, number CRD42020169723.

## Background

### Epidemiology of HCC

Hepatocellular carcinoma (HCC), also known as primary liver cancer, is a disease of global significance due to its high incidence and mortality rates. HCC is the sixth most common cancer worldwide [1]. However, it is the fourth most common cancer in Africa, with differences in prevalence and etiology between North Africa and sub-Saharan Africa (SSA). This variation appears to be due to the varying prevalence of the underlying risk factors for HCC between the different regions [2]. The age-standardized incidence rate (ASIR) of HCC is reported as 6.3/100,000persons/year, 8.6/100,000persons/year, 10.3/100,000persons/year for North Africa, Southern SSA, and Eastern SSA, respectively. Central and Western SSA show the highest incidence of HCC of 16.9/100,000persons/year [3]. Among the top fifteen countries with the highest incidence of HCC in the world, the African continent alone contributes six of these countries [4].

The number of new HCC cases increased from 746,000 in 2012 to 841,080 in 2018, which represents 5% of all cancers globally. Most of these cases were in Asia and Africa [5]. The number of HCC cases is projected to further increase to 1,361,836 by 2040 because of population growth and the slow change in the epidemiology of viral hepatitis [6].

Due to its high mortality rate, HCC is the second most common cause of cancer mortality worldwide [7]. About 782,000 deaths due to HCC occurred in 2018, which represents 8.2% of all cancer-related deaths worldwide. Most of these deaths were recorded in the World Health Organization (WHO) Asian-Pacific and African regions [8].

### Risk factors for HCC

HCC follows a prolonged period of chronic inflammation of the liver as a result of several factors. HBV, HCV, hepatitis D virus (HDV), alcohol abuse, aflatoxin exposure, dietary iron overload, diabetes mellitus, and obesity are the major risk factors for HCC, globally [2]. Viral risk factors especially chronic infections with HBV or HCV are the most common agents related to the development of HCC worldwide in comparison to environmental and behavioral risk factors such as alcohol consumption, obesity, smoking, and exposure to aflatoxins [9]. Globally, 44% of HCC cases are attributed to chronic HBV infection (CHB) while 21% of the cases are attributed to chronic HCV infections [10]. Moreover, 39.1% of HCC deaths have been HBV-related while 29.1% are due to HCV infection, globally [11, 12]. Due to the high attributable fraction of HCC due to CHB, HBV has been coined as the “second-most carcinogenic agent after tobacco” [13].

The incidence of HBV- and HCV-related HCC varies between North Africa and SSA due to variations in the prevalence of infections of the two viruses. HBV contributes 18 and 70%, while HCV is responsible for 60 and 20% of HCC in North Africa and SSA respectively [14]. However, data on the incidence of HCC in most SSA countries are usually either non-existent or inaccurate, which results in a significant underestimation of the tumor in Africa [4, 15].

### Contribution of viral hepatitis to HCC in Africa

About 2 billion people have been infected with HBV globally, of whom 248 million are chronic carriers of the virus and are at an increased risk of liver cirrhosis and HCC [16]. The overall estimated prevalence of CHB on the African continent is 6.1%, with SSA carrying the largest number of 78 million infections [17, 18]. In SSA, Central Africa has the highest CHB prevalence of 9.7% among adults followed by West Africa with an 8.3% prevalence. Eastern SSA and Southern SSA have a 5.5 and 3.8% prevalence of CHB, respectively. North Africa has the lowest prevalence of CHB of 2.8% [19].

An estimated 71.1 million people are currently chronic carriers of HCV worldwide, with about 18 million of these being in Africa and at risk of HCC development [18, 20]. In SSA, the prevalence is estimated at 2.1–3.3% while North Africa has a prevalence of HCV of 2.3–7.7% [19].

HDV is a negative-strand ribonucleic acid virus that requires HBV for its proliferation [21]. Globally, 5% of HBV-infected individuals are co-infected with HDV, with SSA being one of the regions with the highest prevalence (8.39%) [22, 23]. The synergistic effect of HBV/HDV co-infection leads to rapid progression to liver cirrhosis and HCC compared to HBV alone [24, 25].

The epidemiological transition of viral hepatitis-associated HCC for the past four decades in Africa remains poorly described. A clear understanding of these transitions over time and the factors that could influence such changes are key for informing priority areas of intervention for the prevention of viral hepatitis-associated HCC in Africa. This study sought to provide information on the changes in the prevalence of viral hepatitis among HCC patients and the overall effect of the prevalence of viral hepatitis on the incidence of HCC over the past four decades in Africa.

## Materials and methods

The study protocol was designed and reported following Preferred Reporting Items for Systematic Reviews and Meta-Analysis Protocol (PRISMA) guidelines.

### Literature search method

We conducted a systematic literature search per PRISMA guidelines, in MEDLINE (PubMed), Science Direct, Web of Science, Google Scholar, Scopus, and Africa-Wide Information for studies published between January 1980 and Dec 2019 and describing viral hepatitis (HBV, HCV, and HDV), and HCC in Africa. The search terms included (((“viral hepatitis”[Title/Abstract] OR viral hepatitis [Title/Abstract] OR Hepatitis, Viral, Human [Title/Abstract] OR Human Viral Hepatitis [Title/Abstract])) OR (viral hepatitis OR Hepatitis, Viral, Human OR Human Viral Hepatitis [MeSH Terms])) AND (((“hepatocellular carcinoma”[Title/Abstract] OR Carcinoma, Hepatocellular [Title/Abstract] OR Adult Liver Cancer [Title/Abstract] OR Liver [Title/Abstract] OR Hepatoma Cancer AND, Adult [Title/Abstract])) OR (hepatocellular carcinoma OR Carcinoma, Hepatocellular OR Adult Liver Cancer OR Liver OR Hepatoma [MeSH Terms])) AND ((Africa [Title/Abstract]) OR Africa [MeSH Terms]).

**Fig. 1:**
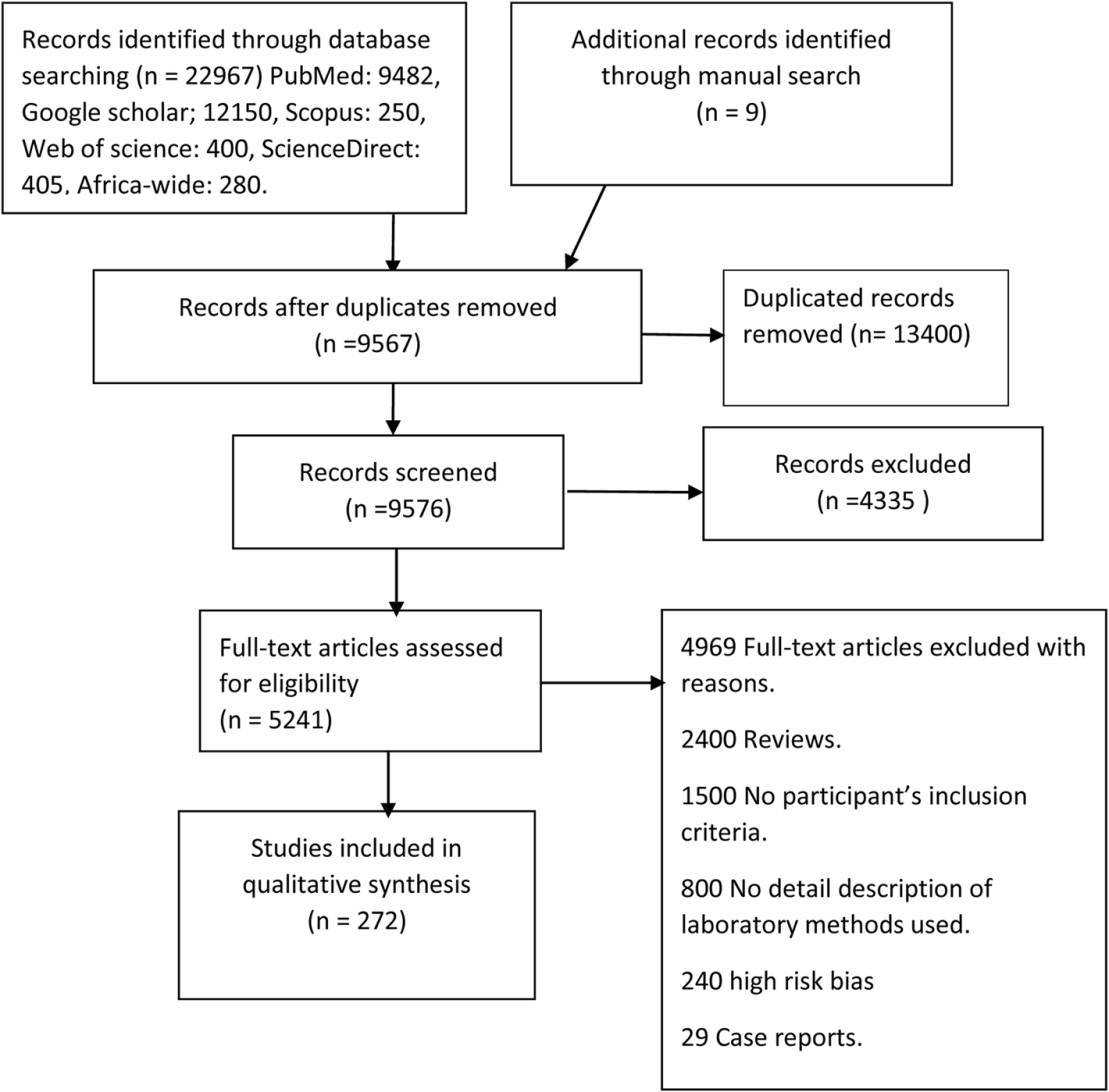
Flow chart of viral hepatitis and HCC articles identified and included in a systematic review in Africa between 1980 and 2019

Titles and/or abstracts were screened to decide the relevance of the studies and the full text of selected studies was retrieved and reviewed. The reference lists of relevant studies were also assessed to find additional studies. The last search was conducted on 31 March 2020, and the search was limited to studies published in English and/or French languages. The languages were limited to English and French because these are the two languages the researchers understand. Duplicated studies and all references were removed and managed by the bibliographic management tool Mendeley Desktop version 1.19.4. The literature search is illustrated in Fig. 1.

### Inclusion/exclusion criteria

All published articles in peer-reviewed journals on viral hepatitis and viral hepatitis-associated HCC on the African continent in the English and French languages between January 1, 1980, and Dec 31, 2019, were assessed for eligibility for inclusion in the study. Studies reporting on the detection of hepatitis B surface antigen (HBsAg), or anti-hepatitis delta antibody (anti-HDV), or HDV RNA, or anti-hepatitis C antibody (anti-HCV), or HCV RNA, with a detailed description of the participants’ selection or inclusion criteria and laboratory methods used, were included in the study. Studies that reported on first-time HCC diagnosis through histology, and/or imaging, and/or biochemical tests were also included. Reviews, case reports/series, editorials, commentaries, and letters were excluded. Research on hepatitis A virus (HAV) and hepatitis E virus (HEV) was excluded because these viruses do not typically cause chronic infections except on few occasions where HEV may cause chronic infections in immunocompromised individuals which can lead to HCC. However, there were no restrictions on the gender of participants, ethnic groups, cultural behavior, or professional activities.

### Data extraction and quality assessment

We extracted details of study publication year, authors name, journal name, the prevalence of HBV, HCV, and HDV, the incidence of HCC, participants demographics (mean age, gender proportions), sample size, country of research, and type of study population. The risk of bias in individual studies was assessed using the Joanna Briggs bias assessment tool for prevalence studies 2017. The quality of studies was assessed by three of the authors (OBD, TGM, and CRT). Discordances were resolved on the quality of a study by discussions. All three authors were involved in the selection of included and excluded articles. This was done to prevent inclusion and exclusion bias.

### Statistical analysis

The calculated prevalence of HBV, HCV, and HDV was determined by calculating the number of positive subjects divided by the total number of subjects screened. The incidence of HCC was determined by the total combined number of new cases divided by the total combined adult population at risk. The studies’ heterogeneity was quantified by I^2^ statistics. We assessed the effect of time on HBV/HCV/HDV prevalence, and the incidence of HCC in the random-effects models by calculating the change in the prevalence of each virus with respect to HCC incidence related to that virus in a decade. The effect of the prevalence of HBV, HCV, and HDV on the incidence of HCC was also assessed using the random-effects model by calculating the percentage (%) change in the incidence of viral hepatitis-related HCC with respect to 1% change in the prevalence of each virus. This model was used to combine the data of primary outcomes, due to the existence of high-level heterogeneity in-between studies. The random effect model determines how much of the variability in the estimates is due to the study differences (study population, age, sex, geographical location, and study design) and how much is due to chance. The pooled prevalence was adjusted for studies heterogeneity and impact of sample size. Box and whisker plots were used to give a visual representation of the changes in the trend of the prevalence of HBV, HCV, and HDV and the incidence of HCC over the period. The statistical significance level was set at *p < 0.05.* All analyses were done using Stata 16.0 (Stata Corp, College Station, Texas, USA).

## Results

### Studies selection and characteristics

**Fig. 2:**
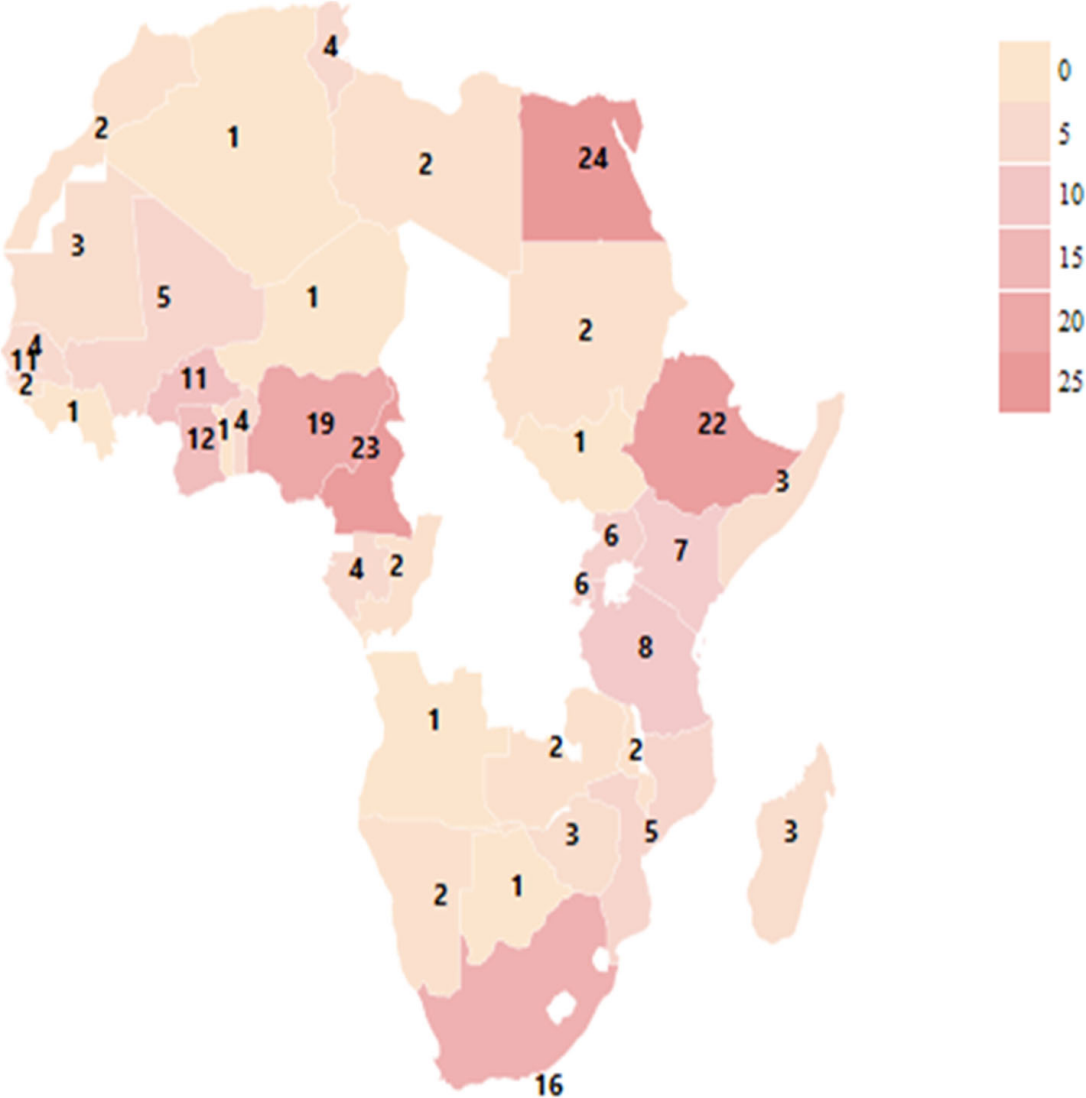
A heat map of Africa showing the distribution of HBV prevalence studies included in the systematic review. The numbers shown represent the number of eligible studies identified in each of the countries. Egypt has the highest number of eligible HBV studies (*n* = 24), followed by Cameroon (*n* = 22), identified, and included in the study. (Map generated from https://geographicheatmap.blogspot.com)

**Fig. 3:**
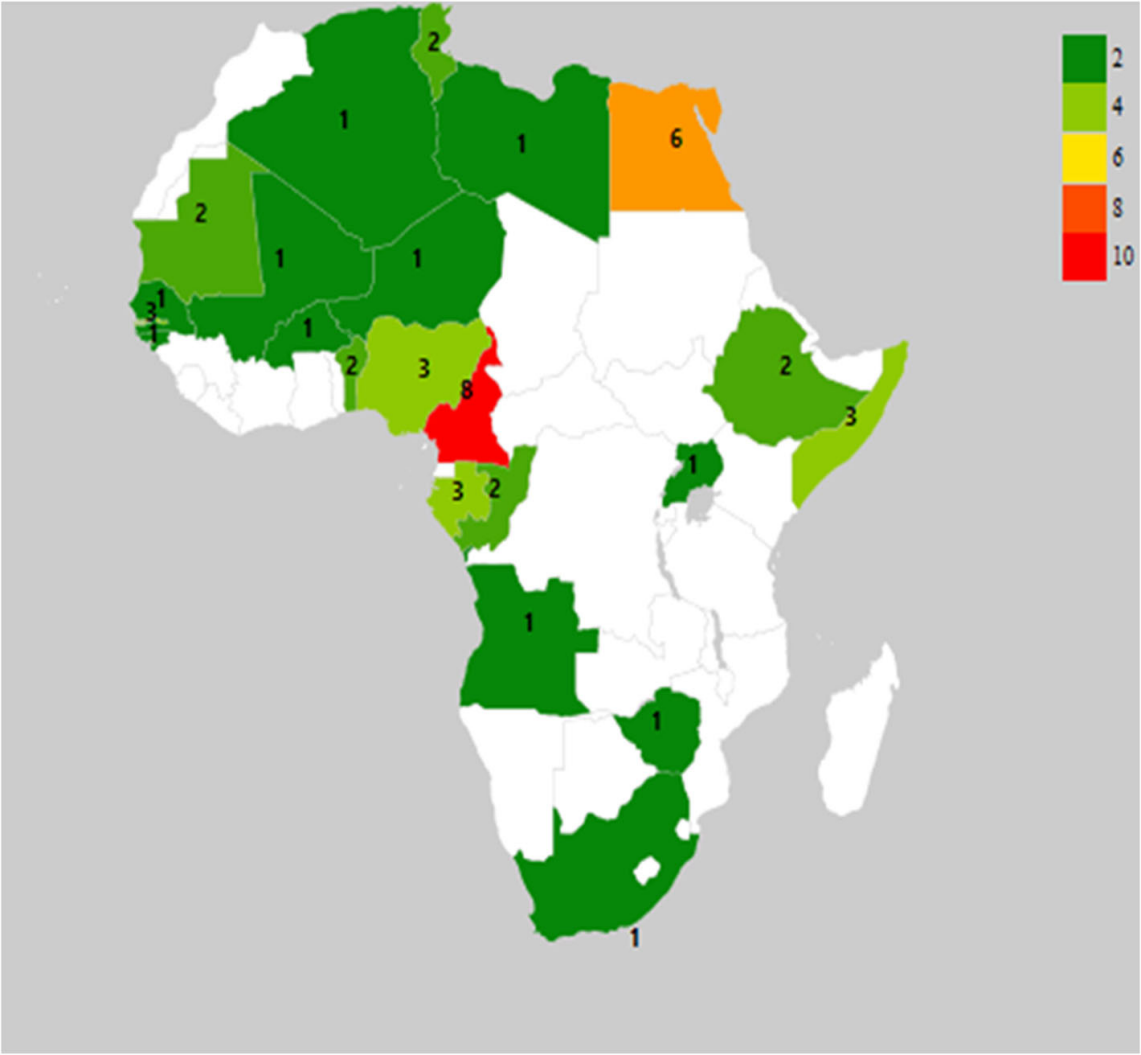
A heat map of Africa showing the distribution of HDV prevalence studies included in the systematic review. Cameroon has the highest number of eligible HDV studies (*n* = 8) while Egypt had the second-highest eligible HDV studies (*n* = 6) identified and included in the study (Map generated from https://geographicheatmap.blogspot.com)

**Fig. 4:**
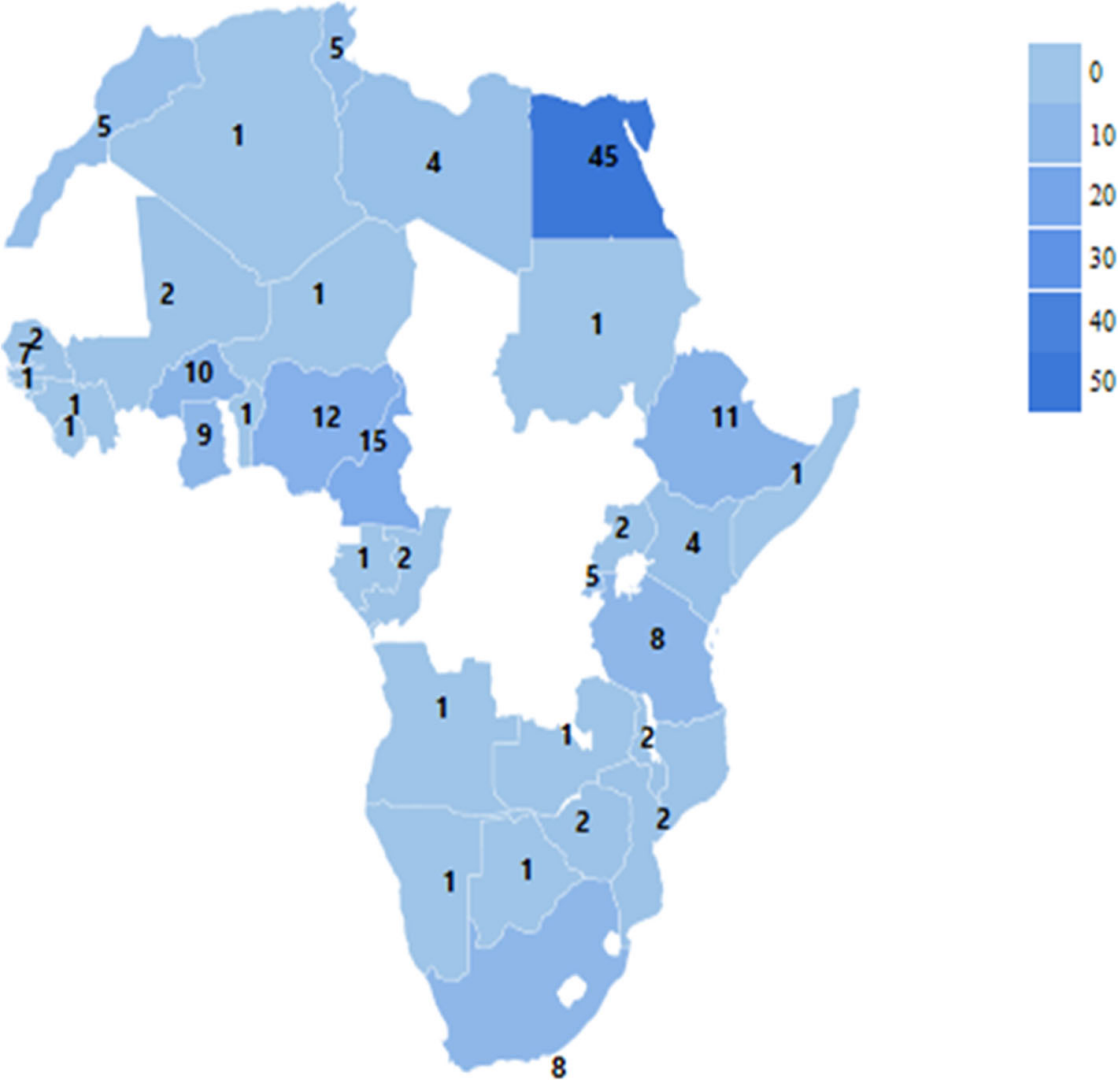
A heat map of Africa showing the distribution of HCV prevalence studies included in the systematic review. The numbers on the map represent the number of eligible studies identified in each country. Egypt has the highest number of eligible studies on HCV (*n* = 45) identified and included in the study (Map generated from https://geographicheatmap.blogspot.com)

Of the 22,967 studies identified and screened, 272 met the study eligibility criteria and were included as shown in Fig. 1. Data on HBV prevalence was available for 244 studies, while 187 studies had HCV prevalence data. Data on HDV was available from 52 studies. HBV and HCV co-infections were reported in 50 studies while 45 studies reported on HCC related to HBV, HCV, and HDV. West Africa had the highest number of studies on the prevalence of HBV and HDV co-infection as shown in Figs. 2 and 3. Egypt in North Africa had the highest number of HCV prevalence (Fig. 4) and viral hepatitis-related HCC studies. The changes (%) in the prevalence of HBV, HCV, HBV/HCV, and HDV, as well as the change in the incidence of HCC, are shown in Table 1. The percentage (%) change in prevalence between each of the viruses (HBV, HCV, and HDV) and HCC are also shown in Table 2. The subregional analyses of the contribution of HBV, HCV, and HDV to HCC incidence in SSA and North Africa are shown in Table 3.

**Table 1:** Change (%) in the prevalence of HBV, HCV, HDV among HCC cases, and the incidence of HCC per decade from 1980 to 2019

Virus and HCC Incidence	Change (%)	95% confidence interval (CI)	***p***-value
**HBV**	**−0.50**	**−0.74 – − 0.25**	**< 0.001**
**HCV**	**− 0.89**	**− 0.49 – 0.31**	**0.66**
**HDV**	**0.01**	**−0.31 – 0.33**	**0.95**
**HBV&HCV co-infection**	**−0.21**	**−0.30 - -0.12**	**< 0.001**
**HCC incidence**	**1.52**	**−4.26 – 7.29**	**0.61**

**Table 2:** Change (%) in the incidence of HBV, HCV, and HDV related HCC per 1% change in the prevalence of each virus

Virus	Change (%)	95% (CI)	***p***-value
**HBV-HCC**	**1.17**	**0.63–1.71**	**< 0.001**
**HCV-HCC**	**0.82**	**0.45–1.18**	**< 0.001**
**HDV-HCC**	**3.34**	**2.44–4.25**	**< 0.001**

**Table 3:** Contribution of HBV, HCV, and HDV to the incidence of HCC in SSA and North Africa

Subregion	HBV % (95% CI)	HCV % (95% CI)	HDV % (95% CI)
**SSA**	**65.8 (39.1–68.3)**	**26.8 (16.9–35.1)**	**14.8 (8.7–18.4)**
**North Africa**	**29.1 (10.3–32.7)**	**46.5 (11.2–48.6)**	**21.2 (12.3–25.6)**

### Prevalence of HBV among HCC cases and incidence of HCC between 1980 and 2019

**Fig. 5:**
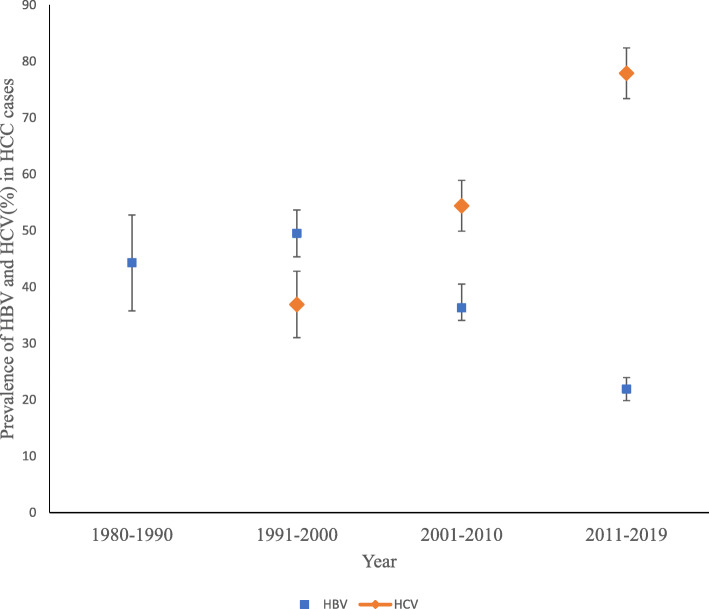
A box and whiskers plot of the prevalence of HBV and HCV in hepatocellular carcinoma patients over four decades in Africa, between 1980 and 2019. HBV (blue box) prevalence among HCC cases decreased significantly while HCV (orange box) prevalence in the HCC cases increased over the study period (1980–2019). The box represents the mean estimate (prevalence), and whiskers correspond to the error margin (HBV = 3.5%, HCV = 3.3%) around the estimated mean

Two hundred and forty-four studies were identified for HBV prevalence and were included in the random-effect meta-regression analysis. From the analysis, the prevalence of HBV in HCC cases changed by − 0.50% (95% confidence interval (CI): − 0.74 – − 0.25) per decade over the four-decade period (Table 1). This change in prevalence was statistically significant (*p < 0.001*) and can be graphically observed in Fig. 5. HBV-related HCC had a change in the incidence of 1.17% (95% CI: 0.63–1.71, *p < 0.001*) for every 1% change in the prevalence of HBV (Table 2). The prevalence of co-infection of HBV and HCV in HCC cases also had a significant change of − 0.21% (95% CI: − 0.30 – − 0.12, *p < 0.001*) per decade over the 4 decades. In the subregional analyses, HBV contributed 65.8% (95% CI: 39.1–68.3) and 29.1% (95% CI: 10.3–32.7) of viral hepatitis-associated HCC in SSA and North Africa respectively (Table 3).

### Prevalence of HCV among HCC cases and incidence of HCC

One hundred and eighty-seven studies reporting on the HCV prevalence were identified and included for the random-effect meta-regression analysis. However, there was no data on HCV-related HCC for the first decade (1980–1990). An increase in the prevalence of HCV among HCC cases was observed over the four-decade period (1980–2019) as shown in Fig. 5, though not statistically significant. HCV prevalence in HCC cases changed by − 0.89% (95% CI: − 0.49 – 0.31, *p = 0.66*) per decade over the four-decade period (Table 1). HCV-related HCC had a change in incidence of 0.82% (95% CI: 0.45–1.18, *p < 0.001*) for every 1% change in the prevalence of HCV on the continent (Table 2). The subregional analyses also showed that HCV contributed 26.8% (95% CI: 16.9–35.1) and 46.5% (95% CI: 11.2–48.6) of viral hepatitis-related HCC cases in SSA and North Africa, respectively.

### Prevalence of HDV among HCC cases and incidence of HCC

**Fig. 6:**
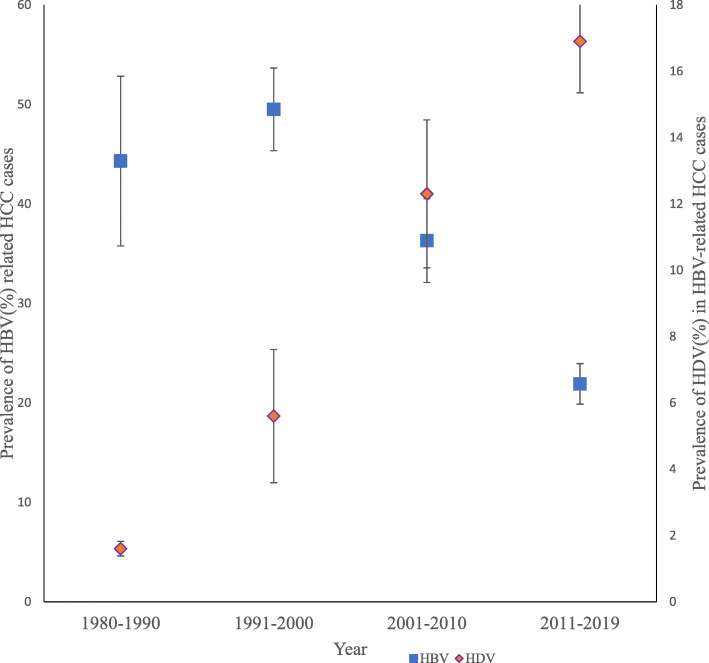
A box and whiskers plot of the prevalence of HDV in HBV-associated hepatocellular carcinoma in Africa between 1980 and 2019. HBV (blue box) prevalence decreased among HCC cases while HDV (orange box) prevalence in the HCC cases increased gradually over the study period (1980–2019). The box represents the mean estimate (prevalence), and whiskers correspond to the error margin (HBV = 3.5%, HDV = 0.48%) around the estimated mean

Fifty-two studies reported on HDV prevalence and were included in the random-effect meta-regression analysis. A gradual increase in HDV among HBV-related HCC cases is observed in Fig. 6. The prevalence of HDV in HCC cases change by 0.01% (95% CI: − 0.31 – 0.33) per decade in the four decades, but this change was not statistically significant (*p = 0.95*) (Table 1). HBV/HDV-related HCC had a change in the incidence of 3.34% (95% CI: 2.44–4.25, *p < 0.000*) for every 1% change in HDV prevalence (Table 2). HBV/HDV co-infection contributed 14.8% (95% CI: 8.7–18.4) and 21.2% (12.3–25.6) of viral hepatitis-related HCC cases in SSA and North Africa respectively in the subregional analysis.

### Incidence of viral hepatitis- associated HCC between 1980 and 2019

**Fig. 7:**
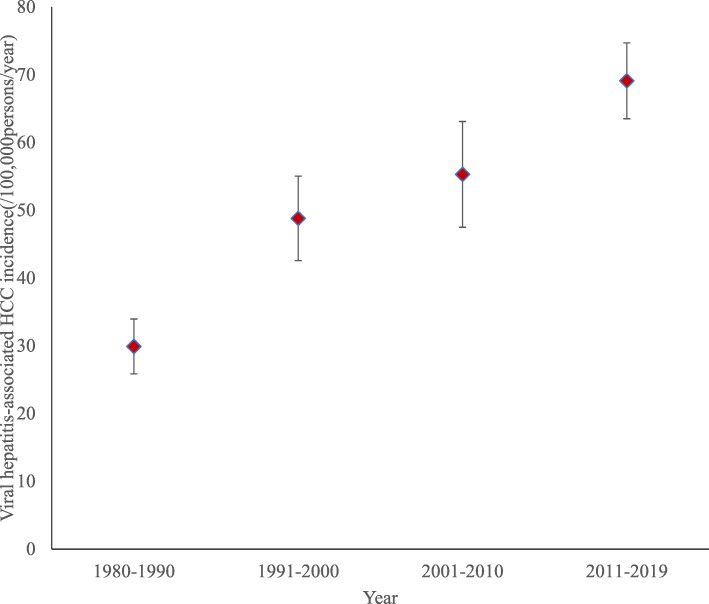
A box and whiskers plot showing the trend of incidence of viral hepatitis-related hepatocellular carcinoma between 1980 and 2019. Viral hepatitis-related hepatocellular incidence (blue box) increased over the study period covering 1980 to 2019. The incidence was 29.9/100,000person/year, 48.8/100,000persons/year, 55.3/100,000persons/year and 69.5/100,000person/year for the first (1980–1990), second (1991–2000), third (2001–2010) and the fourth (2011–2019) decades, respectively. The box represents the mean estimate (prevalence) and whiskers correspond to the error margin (3.2%) around the estimated mean

Forty-five studies on viral hepatitis-related HCC incidence were included in the analysis. The incidence of viral hepatitis-related HCC increased steadily over the four decades (1980–2019) as indicated in Fig. 7. The incidence of viral hepatitis-related HCC was 29.9/100,000persons/year for the period 1980–1990, 48.8/100,000persons/year for the period 1991–2000, 55.3/100,000persons/year for the period 2001–2010, and 69.5/100,000persons/year for the period 2011–2019. The median onset age of HCC was 47 years and the male to female ratio among HCC cases was 3:1. In the random-effect meta-regression analysis, we found that the incidence of viral hepatitis-related HCC changed by 1.52% (95% CI: − 4.26 – 7.29) each decade over the four-decade period but this was not statistically significant *(P = 0.61)*. In comparing the prevalence of HBV and HCV among HCC cases over the 40 years, there was a slight decline in HBV prevalence while HCV prevalence increased.

## Discussion

Viral hepatitis is largely responsible for the incidence of HCC across the globe. The prevalence of chronic viral hepatitis (mostly HBV and HCV) has been strongly associated with the incidence of HCC in many studies across the world [2, 5, 18]. Therefore, it is important to understand the trends of the prevalence of HBV and HCV on the African continent in making policies and recommendations for the prevention of viral hepatitis-related HCC.

### Effect of HBV

The number of studies on viral hepatitis-associated HCC began to increase from the 1990s. Increased diagnosis capacity and funding for viral hepatitis research on the continent could be responsible for this observation. Studies on HBV were fairly distributed evenly across the continent while most studies on HCV came from Egypt. HBV contributes the highest number of HCC cases in the world, especially in Africa where CHB is highly endemic [9, 12]. The risk of developing HBV-related HCC is 223 times high in patients with CHB compared to non-infected individuals [26]. In the current study, the prevalence of HBV was found to decline among HCC cases between 1980 and 2019. This finding was consistent with studies conducted in Asia [27]. The decline in HBV prevalence is likely a direct result of the increased coverage of childhood HBV immunization programs implemented across the African continent [28, 29]. Childhood HBV vaccination has been effective in reducing the infection rates of HBV worldwide [30] which results in fewer people developing CHB that progresses to HCC. Although the decline in HBV-related HCC cases is a remarkable achievement for the African continent, there is still a need for improvement in HBV birth dose vaccine coverage across the continent and treatment for those already infected. There are still some countries that have not fully implemented the HBV birth dose vaccine in their childhood immunization schedules [31]. HBV birth dose immunization is important because perinatally-acquired HBV infections are likely to progress to CHB, leading to the development of HCC [32]. Increased political commitment from African governments to provide resources for the implementation of the HBV birth dose immunization programs is required. This will help in the complete elimination of CHB and its sequelae that includes liver cirrhosis and HCC [9]. Other strategies that could also contribute to the control of the African HBV epidemic include increased awareness of HBV among the general population, improved safety in blood transfusions, safe tattooing, and hygienic traditional scarification [33].

### Effect of HCV

HCV is the second major risk factor for HCC in Africa after HBV. Our findings showed an increase in the prevalence of HCV among HCC cases, and this is consistent with similar findings in the global liver cancer incidence by Lin et al. [12]. This increase may be attributed to the inadequate awareness of transmission routes of the virus and its complications, the unavailability of a vaccine, and limited access to direct-acting antiviral drugs (DAAs) [18]. Studies conducted in Ghana and Egypt among barbers and HCV-infected patients respectively demonstrated limited knowledge of HBV and HCV among the studied populations [34, 35]. For an improved understanding of the epidemiology of HBV and HCV-induced HCC in Africa, there is a need for screening for viral infections, especially in high-risk populations such as prison inmates, commercial sex workers, people who inject drugs, and men who have sex with men, because early detection of the infection has better treatment outcomes and helps to prevent HCC development [33]. With reduced costs for testing and treatment, improved access to antiviral drugs (DAAs), increased awareness of the virus and its complications among the general population, and education and/or support for IDUs, the burden of HCV and HCV-related HCC will decrease significantly on the African continent [36].

### Effect of HDV

The prevalence of HDV in HBV-infected individuals increased gradually over the study period, although this increment was not statistically significant in the random-effect analysis. The increased prevalence of HDV is of concern owing to the synergistic effect of HDV on the progression of HBV-related HCC [37]. The increasing prevalence of HDV among HBV infected individuals means these people are at increased risk of HCC compared to HBV mono-infected individuals. As indicated in our results, decreasing the prevalence of HDV by 1% decreases the incidence of HBV/HDV-related HCC by 3.34 times. The increasing prevalence of HDV underlines the need to prevent HBV infections since HDV requires HBV for its replication and carcinogenic activities [24, 25]. Our subregional analyses further confirm this point because the increasing prevalence of HDV has resulted in increased HDV related HCC in both SSA and North Africa compared to similar findings in [23, 25].

From the analysis, there was a steady increase in the incidence of viral hepatitis-related HCC between 1980 and 2019. Our findings showed that the incidence of viral hepatitis-associated HCC increased by 1.52% each decade, but this was not statistically significant. The incidence could be higher and probably statistically significant if African countries had a better screening and diagnostic capacity for HCC, proper data capturing systems at cancer registries, and adequate cancer registries. The increasing trend in the incidence of viral hepatitis-associated HCC could be a result of the increasing prevalence of HCV on the African continent over the last 40 years. It could also be due to the limited access to antiviral drugs against HBV and HCV; therefore, a significant number of the chronic carriers of HBV and HCV in the population have an increased risk of HCC and may eventually develop HCC later in life.

Despite the prevailing viral risk factors on the continent, there are other factors responsible for the increasing incidence of viral hepatitis-induced HCC in Africa. These factors include inadequate trained professionals such as hepatologists and radiologists in Africa [38] and fewer resources committed to the fight against viral hepatitis-related HCC by African governments over the years [15]. The selective attention due to limited resources committed by African governments could explain the mixed picture of viral contribution to HCC found in the subregional analyses. According to Bahri et al. [39] and Ndom [14], HBV contributed 70 and 18% of HCC in SSA and North Africa respectively while HCV contributed 20 and 60% of HCC in SSA and North Africa, respectively. However, in the current study, we found that HBV contributed 65.8 and 29.1% of viral hepatitis-related HCC in SSA and North Africa, respectively. HCV also contributed 26.8 and 46.5% of viral hepatitis-related HCC cases in SSA and North Africa, respectively. The decreased-increased scenario of HBV and HCV-related HCC in Africa confirms the unequal attention and resources committed to the fight against these two viruses. It also calls for the sharing of policy-implementation strategies between African governments. African governments must work in cooperation and commit enough resources towards the elimination of viral hepatitis and HCC on the continent.

However, our findings could be as result of improved testing capabilities, changes in healthcare practices, policies, and infrastructure rather than epidemiological changes because we do not have data on the overall incidence of HCC (viral and non-viral) in Africa. Therefore, these changes in HBV/HCV/HDV prevalence could be in part due to changes in how frequently these viruses are tested for in HCC patients over time.

The median age for the onset of HCC was 47 years and the male to female ratio in HCC cases was 3:1. These findings were consistent with another study by Kirk et al. in the Gambia [40]. The current age of onset of HCC in Africa (47 years) is the youngest in the world compared with other regions such as Japan (69 years), Europe (63 – 65 years), North America (62 years), Korea (57 years), and China (55 – 59 years) [1]. The early onset of HCC in Africa would result in loss of labor force and reduced productivity, resulting in an increased economic burden on the continent.

In contrast to the findings of the current study, studies conducted in Asia found a declining trend of HBV and HCV-related HCC over a similar study period. Factors such as improved HBV vaccine uptake, increased access to both HBV and HCV antiviral drugs, and improved diagnostic tools were stated as being responsible for the decline in the incidence of HCC [27]. To significantly reduce the incidence of viral hepatitis-associated HCC in Africa, there should be an implementation of effective screening and preventive programs for chronic viral hepatitis in Africa and increased access to antiviral treatment for HBV and HCV. Our findings reaffirm the need for increased efforts in the prevention of HBV and HCV in the fight against HCC on the African continent. There is also a need for the implementation of effective surveillance systems that will aid in the prevention, early diagnosis, and treatment of HCC in Africa. Efforts should be made to minimize the other risk factors such as alcohol abuse, diabetes, aflatoxin exposure, smoking, and obesity as these factors can hasten the development of viral hepatitis-associated HCC. These factors can also be confounding factors in the disease (HCC) development process, and therefore can affect the evaluations of interventions for the prevention of viral hepatitis-associated HCC in Africa. Human immunodeficiency virus (HIV) could also have an impact on the trends of HBV and HCV, and the development of HCC in Africa. With HIV, HBV infections are likely to become chronic, therefore the development of HCC may occur quicker in HIV patients with chronic viral hepatitis than non-HIV infected individuals [41].

### Limitations

The current study includes only articles published in the English and French languages; this left out quality articles that were published in other languages in Africa. Data on the overall incidence of HCC in Africa was not collected due to time constraints, so a comparison between total HCC cases and viral hepatitis-related HCC could not be done. Furthermore, many studies conducted in the 1980s did not clearly describe laboratory procedures, and when we contacted the authors, most of them did not respond. These studies were left out of this systematic review. Including them could probably have a different outcome in the results. Moreover, most of the studies were conducted over a longer period and therefore the year of publication did not necessarily reflect the incidence for that year. Also, none of the data was from a cancer registry of any country and this shows the fragmentation in the data gathering process in Africa, which could influence our results. Finally, since we could not look at the overall incidence of HCC in Africa (we looked at only viral hepatitis-related HCC) our findings could be as a result of improved testing capabilities in Africa rather than actual changes in the frequency viral hepatitis-related HCC. This is a limitation we would explore in our next research.

## Conclusion

Viral hepatitis-associated HCC is still a major public health problem in Africa; HBV and HCV are responsible for a larger proportion of all HCC cases in Africa. Although efforts have been made by different role players including individual governments as well as regional and international health organizations to minimize the infection rates of HBV and HCV in Africa, the prevention of HBV-related HCC through HBV vaccination is yet to achieve maximum success. It is also clear from our findings that healthcare systems in Africa must prepare for the increasing number of patients with hepatitis-related HCC despite the implementation of prevention programs. Therefore, there is a need for increased and sustained efforts towards the elimination of HBV and HCV as public health concerns. To eliminate viral hepatitis and its sequelae such as cirrhosis and HCC, improved access to antiviral drugs for individuals infected with HBV and HCV, implementation of control and preventive measures for HBV and HCV, and increased funding for research into viral hepatitis for the African continent must be given priority by health policymakers.

## Data Availability

The datasets used and analyses during the current study are available from the corresponding author on reasonable request.

## Abbreviations

HCC: Hepatocellular Carcinoma
HBV: Hepatitis B virus
HCV: Hepatitis C virus
HDV: Hepatitis D virus
HAV: Hepatitis A virus
HEV: Hepatitis E virus
SSA: sub-Saharan Africa
WHO: World Health Organization
HIV: Human Immunodeficiency Virus
DAAs: Direct-Acting Antiviral drugs
CHB: Chronic Hepatitis B
